# Association and Comparative Analysis of Four Inflammatory Indices With 90‐Day Outcomes in Acute Ischemic Stroke Patients Treated With Intravenous Thrombolysis

**DOI:** 10.1002/brb3.71433

**Published:** 2026-05-05

**Authors:** Xinping Bai, Xiangyu Wu, Zhuo Cai, Hao Cheng, Juluo Chen

**Affiliations:** ^1^ Department of Neurology Fuyang Hospital Affiliated to Anhui Medical University Anhui People's Republic of China; ^2^ Department of Neurology Fuyang People's Hospital Anhui People's Republic of China

**Keywords:** ischemic stroke, NLR, outcome, PLR, SII, SIRI, thrombolytic therapy

## Abstract

**Objective:**

To investigate and compare the associations of the systemic immune‐inflammation index (SII), the systemic inflammation response index (SIRI), the platelet‐to‐lymphocyte ratio (PLR), and the neutrophil‐to‐lymphocyte ratio (NLR) with 90‐day functional outcomes in AIS patients treated with IV rt‐PA., with particular attention to their nonlinear relationships and threshold effects.

**Methods:**

Note that 503 AIS patients who received intravenous thrombolysis with alteplase (rt‐PA) were consecutively enrolled, and clinical information along with laboratory data were collected. ROC curve analysis was conducted to determine the optimal cut‐off value of SII, PLR, NLR, and SIRI. Multivariate analysis was included for variables with *p* < 0.05 in univariate analysis. The restricted cubic spline (RCS) curve illustrates the nonlinear relationship between SII, PLR, NLR, SIRI, and the 90‐day unfavorable outcomes.

**Results:**

A total of 503 patients were included. According to multivariate logistic regression analysis, admission NIHSS scores (OR = 1.156, 95% CI: 1.049–1.274), albumin (OR = 0.875, 95% CI: 0.774–0.989), and SII (OR = 1.004, 95% CI: 1.001–1.006) were independent indicators of unfavorable outcomes at 90 day after intravenous thrombolysis (*p* < 0.05). The ROC analysis showed that an SII cutoff value of 1268.00 could predict poor 90‐day outcomes with a sensitivity of 61.58% and a specificity of 92.02%. The AUC was 0.764 (95% CI 0.677–0.852, *p* < 0.05). Pairwise comparison using the DeLong test revealed that SII and NLR demonstrated comparable discriminative ability (*p* = 0.392), and both significantly outperformed PLR (SII vs. PLR: *p* = 0.001; NLR vs. PLR: *p* = 0.006). No significant difference was detected between SII and SIRI (*p* = 0.709) or between NLR and SIRI (*p* = 0.211). SII showed the most consistent independent association in multivariable analysis (OR = 1.004, 95% CI: 1.001–1.006). The RCS curve illustrates the nonlinear relationship between SII, PLR, NLR, SIRI, and the 90‐day unfavorable outcomes.

**Conclusions:**

Among the inflammatory indices studied, SII showed the strongest association with 90‐day unfavorable outcomes in this cohort. Our findings suggest a potential role of systemic inflammation in patient prognosis post‐thrombolysis, warranting further prospective validation.

## 1 Introduction

Cerebrovascular disease remains a leading cause of mortality and long‐term disability worldwide (Q. Ma et al. 2021); the number of new cases is increasing each year. Over 2.4 million newly diagnosed strokes occur in China annually, with a mortality rate of 22.3% (Ren et al. 2017; Y. J. Wang et al. 2020). Of these patients, 87% have ischemic stroke (T. Zhu et al. 2021). Currently, the most effective pharmacological treatment for acute ischemic stroke is thrombolysis with alteplase within 4.5 h (Powers et al. 2019), aiming to restore cerebral blood flow and mitigate neurological deficits. However, although there have been improvements in thrombolytic therapy, there are still variations in clinical outcomes, and many patients continue to experience poor outcomes. Several observational studies (Herath et al. 2021; Çetiner et al. 2018; Tang et al. 2021) have shown that intravenous thrombolysis significantly reduces disability between 3 and 6 months. Many factors that impact clinical outcomes have been extensively researched. Growing attention has been given to the role of systemic inflammation in the pathophysiology and prognosis of ischemic stroke (Pagram et al. 2016).

Systemic inflammation processes are closely associated with cerebral blood flow autoregulation, blood‐brain barrier permeability, and endothelial dysfunction, which may influence the development of ischemic stroke (Evans et al. 2021). Recently, four novel inflammatory markers, the systemic immune‐inflammation index (SII), the system inflammation response index (SIRI), the platelet‐to‐lymphocyte ratio (PLR), and the neutrophil‐to‐lymphocyte ratio (NLR) composed of platelet and three subtypes of WBC, can suitably reflect the immune status (F. Ma et al. 2023), and proposed associations exist between these factors and poor stroke outcomes at admission (Weng et al. 2021). But these indexes have no wide application to predict functional outcomes in stroke patients. While individual markers like NLR and PLR have been studied in stroke cohorts, including some receiving thrombolysis (Gong et al. 2021, 2019), direct comparisons of the prognostic potential of SII, PLR, NLR, and SIRI specifically within a homogeneous cohort treated with intravenous alteplase are limited. Furthermore, the optimal timing of assessment and their independent value relative to established clinical indicators remain unclear. Given the heterogeneity of ischemic stroke, stroke etiology—classified by the TOAST system—can significantly influence risk factor profiles, initial severity, and long‐term outcomes. Therefore, this study aimed to explore and compare the associations of these four readily available inflammatory indices, measured early after admission, with 90‐day functional outcomes in AIS patients treated with IV rt‐PA.

## 2 Methods

### 2.1 Patients and Participants

**FIGURE 1:**
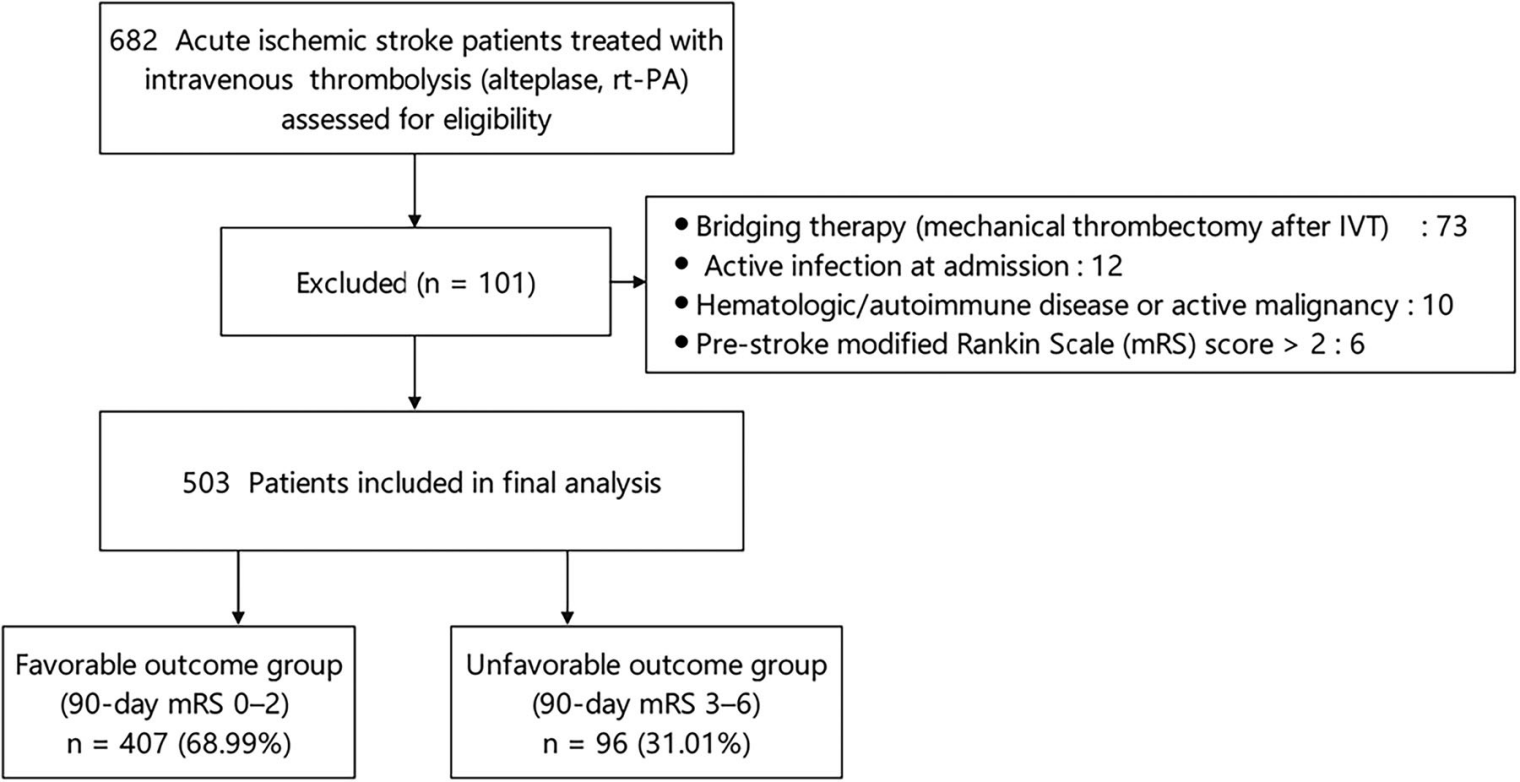
The screening process for the enrolled cases.

This study is a single‐center, retrospective, observational analysis. Patients with acute ischemic stroke who received intravenous thrombolysis with alteplase (rt‐PA) were enrolled from the stroke center of Fuyang People's Hospital from May 2020 to December 2024. The criteria are as follows: (1) aged ≥ 18 years old, who met the criteria for intravenous thrombolysis in acute ischemic stroke and completed the procedure. (2) premorbid mRS ≤ 2. (3) Patients or their family members have signed the informed consent. Exclusion criteria consisted of (1) patients treated with bridging therapy; (2) patients with blood system diseases, autoimmune diseases, or malignant tumors; (3) patients with acute or chronic infections; and (4) patients with incomplete clinical data. This study was approved by the Research Ethics Committee at Fuyang People's Hospital ([2022]159). The screening process is shown in Figure 1.

### 2.2 Data Acquisition

Each participant underwent a standardized assessment to record their demographic characteristics. which included factors such as gender, age, National Institutes of Health Stroke Scale (NIHSS) score, modified Rankin Scale (mRS) scale, vascular risk factors (diabetes, hypertension, coronary heart disease, Atrial fibrillation, prior stroke, and history of alcohol consumption or smoking), etc. Upon admission, the severity of the ischemic stroke was assessed using the NIHSS score. During follow‐up, the modified Rankin score (mRS) was used to evaluate the 90‐day outcomes, with a score of 0–2 indicating a favorable outcome, while a score of 3–6 indicates an unfavorable outcome (Shi et al. 2019). A blinded neurologist evaluated all patient outcomes without clinical data.

### 2.3 Definition of Inflammatory Indicators

Fasting venous blood samples were collected in the emergency department immediately after admission and prior to the initiation of intravenous alteplase thrombolysis. The time from symptom onset to blood sampling was recorded. Laboratory parameters including neutrophils, lymphocytes, platelets, monocytes, and albumin were measured from these pre‐thrombolysis samples. We calculated the SII, PLR, NLR, and SIRI according to the following equations: SII = (neutrophil count × platelet count)/lymphocyte count; PLR = platelet count/lymphocyte count; NLR = neutrophil count/lymphocyte count; and SIRI = (neutrophil count × monocyte count)/lymphocyte count.

### 2.4 Statistical Analysis

Continuous variables are presented as the means ± standard deviations (SDs) or medians (interquartile ranges, IQRs) and were compared using *t*‐tests or Wilcoxon rank‐sum tests, whereas categorical data were compared with chi‐square tests. Variables that exhibited significant differences in univariate analysis were included in multivariable logistic regression models to identify potential risk factors. Two‐sided *p* values < 0.05 were considered statistically significant. Given that SII, PLR, NLR, and SIRI share overlapping cellular components, we assessed multicollinearity in the multivariate logistic regression model using variance inflation factors (VIF). A VIF >  5 was considered indicative of significant collinearity. The receiver operating characteristic (ROC) curve was used to test each indicator's ability to discriminate for a 90‐day prognosis, and the optimal cutoff value was identified based on the greatest sum of sensitivity and specificity. Restricted cubic splines (RCS) with four knots (at the 5th, 35th, 65th, and 95th percentiles) were used to model potential nonlinear relationships between inflammatory indices. The nonlinearity was tested using a likelihood‑ratio test comparing the RCS model with a linear model. Statistical analyses were performed using IBM SPSS 27.0, GraphPad Prism 10.0, and R version 4.3.3.

## 3 Results

### 3.1 Patient Characteristics

The clinical and demographic data for the 2 groups are presented in Table 1. A total of 503 patients who received intravenous thrombolysis for acute ischemic stroke were included in this study. Of these patients, 347 (68.99%) were male and 156 (31.01%) were female. and there was a statistical difference in gender between the two groups. Compared to the favorable outcomes group, the unfavorable Outcomes group had higher age, Atrial fibrillation, admission NIHSS‐scores, uric acid, neutrophil counts, SII, PLR, NLR, and SIRI and lower albumin, the differences were statistically significant. In terms of stroke etiology, the group with favorable outcomes had the highest proportion of SAA, while the group with unfavorable outcomes had the highest proportion of LAA; the difference between the two groups was statistically significant (*p* < 0.001).

**TABLE 1:** Comparison of baseline characteristics in patients with favorable and unfavorable 90‐day outcomes.

Characteristics	Favorable outcomes (*n* = 407)	Unfavorable outcomes (*n* = 96)	*χ* ^2^/*z*	*p*‐value
Gender			4.239	0.04
Male	295 (72.5)	53 (55.2)		
Female	112 (27.5)	43 (44.8)		
Age	62.64 ± 12.97	68.13 ± 13.06	−2.349	0.020
Smoking	180 (44.2)	33 (34.4)	1.253	0.263
Alcohol user	183 (45.0)	30 (31.2)	2.290	0.130
Atrial fibrillation	27 (6.6)	18 (18.8)	5.150	0.023
Hypertension	247 (60.7)	58 (60.4)	0.001	0.981
Diabetes	90 (22.1)	28 (29.1)	0.810	0.368
Ischemic heart disease	40 (9.8)	20 (20.8)	3.700	0.054
Dyslipidemia	119 (29.2)	25 (26.0)	0.147	0.701
Stroke history	90 (22.1)	20 (20.8)	0.019	0.890
Pre‐thrombolysis systolic pressure	148.78 ± 19.47	152.45 ± 22.65	−1.013	0.312
Admission NIHSS score, median (IQR)	4 (2–6)	8 (5–13)	−5.318	0.000
DNT (min), median (IQR)	46.00 (37.75–60.00)	49.00 (40.00–68.50)	−0.947	0.344
fibrinogen (g/L)	3.22 (2.81–3.73)	3.46 (2.97–3.90)	−1.405	0.160
fasting glucose (mmol/L)	6.70 (5.70–8.6)	6.58 (5.49–9.28)	−0.146	0.884
Uric acid (µmol/L)	308 (251–359.35)	280 (210–328.75)	−1.960	0.050
PLT (×109/L)	191 (157–224)	197 (174.75–236.5)	−0.924	0.355
Neutrophil × 109/L, median (IQR)	4.21 (3.27–5.34)	5.98 (4.60–7.66)	−4.072	0.000
Lymphocyte × 109/L, median (IQR)	1.72 (1.29–2.27)	1.51 (1.23–1.96)	−1.471	0.141
mononuclear × 109/L, median (IQR)	0.39 (0.32–0.52)	0.39 (0.31–0.57)	−0.170	0.865
Albumin	42.67 ± 3.78	40.67 ± 3.41	2.988	0.003
BMI	24.8 (22.23–26.70)	23.80 (21.90–26.00)	−1.490	0.136
SII	418.55 (292.45–687.44)	699.53 (391.93–1380.00)	−3.218	0.001
PLR	108.75 (84.86–151.35)	135.05 (93.67–195.12)	−2.007	0.045
NLR	2.24 (1.66–3.47)	3.67 (2.20–5.86)	−3.441	0.001
SIRI	0.95 (0.60–1.37)	1.51 (0.98–2.38)	−3.013	0.003
Stroke subtype (TOAST)			27.043	<0.001
LAA	102 (25.1)	43 (44.8)		
SAA	284 (69.8)	30 (31.3)		
CE	10 (2.5)	18 (18.8)		
SAO	3 (0.7)	2 (2.1)		
SUE	89 (2.0)	3 (3.1)		

Abbreviations: CE, cardio‐embolism; LAA, larger artery atherosclerosis; NIHSS, National Institutes of Health Stroke Scale; NLR, neutrophil‐lymphocyte ratio; PLR, platelet‐lymphocyte ratio; SAA, small‐artery atherosclerosis; SAO, small‐artery occlusion; SII, systemic immune‐inflammation index; SIRI, system inflammation response index; SUE, stroke of undetermined etiology.

### 3.2 Levels of Composite Inflammatory Indicators With Different Outcomes

**FIGURE 2:**
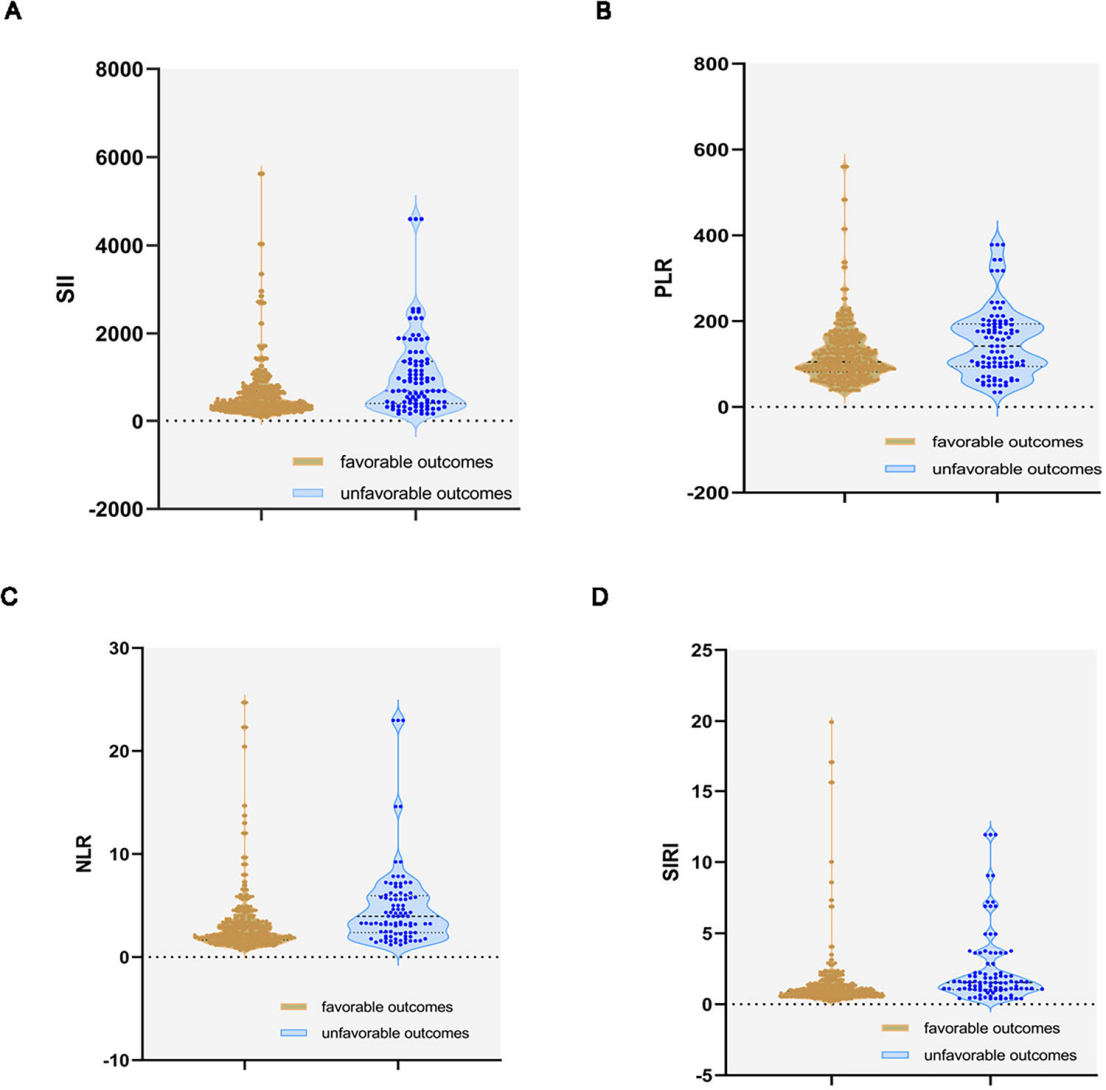
Boxplots of the SII, PLR, NLR, and SIRI showing the distribution in the favorable Outcomes group (*n* = 407) and unfavorable Outcomes group (*n* = 96). NLR, neutrophil‐to‐lymphocyte ratio; PLR, platelet‐to‐lymphocyte ratio; SII, systemic immune‐inflammation index; SIRI, systemic inflammation response index.

Violin plots of the SII, PLR, NLR, and SIRI show the distribution in the favorable Outcomes group (*n* = 407) and unfavorable Outcomes group (*n* = 96) (see Figure 2). Patients in the favorable Outcomes group had lower SII, PLR, NLR, and SIRI levels than in the unfavorable Outcomes group (*p* < 0.05).

### 3.3 Prognostic Value of SII, PLR, NLR, and SIRI in AIS Patients With Intravenous Thrombolysis

**FIGURE 3:**
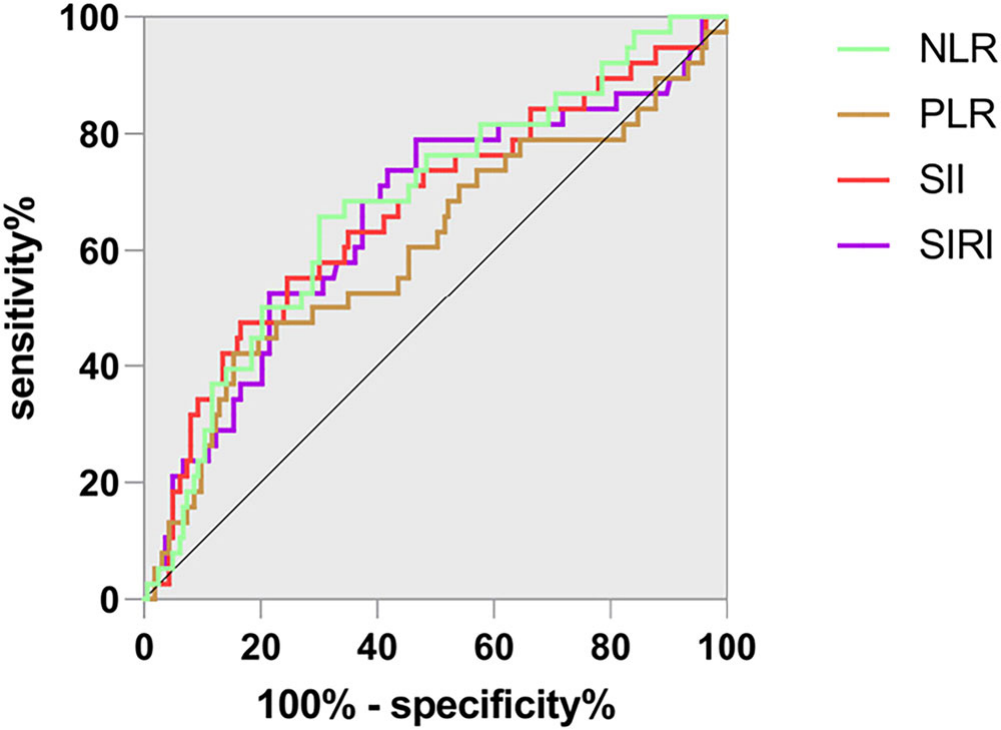
ROC curves of the NLR, PLR, SII, and SIRI for predicting unfavorable outcomes. NLR, neutrophil‐to‐lymphocyte ratio; PLR, platelet‐to‐lymphocyte ratio; SII, systemic immune‐inflammation index; SIRI, systemic inflammation response index.

The ROC analysis showed that an SII cutoff value of 1268.00 could predict poor 90‐day outcomes with a sensitivity of 61.58% and a specificity of 92.02% (Figure 3; Table 2). The AUC was 0.764 (95% CI 0.677–0.852, *p* < 0.05). Similarly, a PLR cutoff value of 171 could differentiate a 90‐day unfavorable outcome with a sensitivity of 42.11%, a specificity of 84.66%, and an AUC of 0.648 (95% CI 0.540–0.757; *p* < 0.05). An NLR cutoff value of 3.13 was found to have a sensitivity of 65.79% and specificity of 69.94% with an AUC of 0.717 (95% CI 0.621–0.813, *p* < 0.05). Lastly, a SIRI cutoff value of 0.978 had a sensitivity of 78.95% and specificity of 53.37%, with an AUC of 0.709 (95% CI 0.608–0.810, *p* < 0.05).

**TABLE 2:** AUC in predicting unfavorable outcomes at 90 days after intravenous thrombolysis.

	AUC (95% CI)	Cutoff‐point	Sensitivity	Specificity	PPV (%)	NPV (%)	*p*‐value
SII	0.764 (0.677–0.852)	1268.00	61.58	92.02	65.3	90.8	<0.001
PLR	0.648 (0.540–0.757)	171	42.11	84.66	39.1	86.4	0.04
NLR	0.717 (0.621–0.813)	3.13	65.79	69.94	33.5	89.7	<0.001
SIRI	0.709 (0.608–0.810)	0.978	78.95	53.37	28.4	91.5	<0.001

Abbreviations: AUC, area under the curve; CI, confidence interval; NLR, neutrophil‐to‐lymphocyte ratio; NPV, negative predictive value; PLR, platelet‐to‐lymphocyte ratio; PPV, positive predictive value; SII, systemic immune‐inflammation index; SIRI, systemic inflammation response index.

Using the optimal cut‐off of 1268.00, SII yielded a PPV of 63.5% and an NPV of 91.2% for predicting unfavorable outcomes, based on the observed prevalence of 19.1%. Detailed predictive parameters for all four indices are presented in Table 2.

### 3.4 Pairwise Comparison of Discriminative Ability: DeLong Test

To determine whether the observed differences in AUCs among the four inflammatory indices were statistically significant, we performed pairwise DeLong tests for correlated ROC curves (Table 2). SII demonstrated a significantly higher AUC compared to PLR (AUC difference = 0.058, 95% CI: 0.024–0.093, *p* = 0.001). However, no statistically significant difference was detected between SII and NLR (AUC difference = −0.013, 95% CI: −0.044–0.017, *p* = 0.392) or between SII and SIRI (AUC difference = 0.008, 95% CI: −0.035–0.052, *p* = 0.709). NLR also showed significantly better discriminative ability than PLR (AUC difference = 0.072, 95% CI: 0.020–0.123, *p* = 0.006). No other pairwise comparisons reached statistical significance (all *p* > 0.05; Table 3).

**TABLE 3:** Pairwise comparison of area under the ROC curves using the DeLong test.

Comparison	AUC difference	Standard error⁠	95% CI	*Z*	*p*‐value
SII—PLR	0.058	0.255	0.058–0.255	3.334	0.001
SII—NLR	−0.013	0.246	−0.013–0.246	−0.855	0.392
SII—SIRI	0.008	0.252	0.008–0.252	0.373	0.709
PLR—NLR	−0.072	0.252	−0.072–0.252	−2.741	0.006
PLR—SIRI	−0.05	0.258	−0.05–0.258	−1.459	0.145
NLR—SIRI	0.022	0.247	0.022–0.247	1.251	0.211

### 3.5 Association of SII, PLR, NLR, and SIRI With 90‐Day Outcomes in AIS Patients With Intravenous Thrombolysis

**FIGURE 4:**
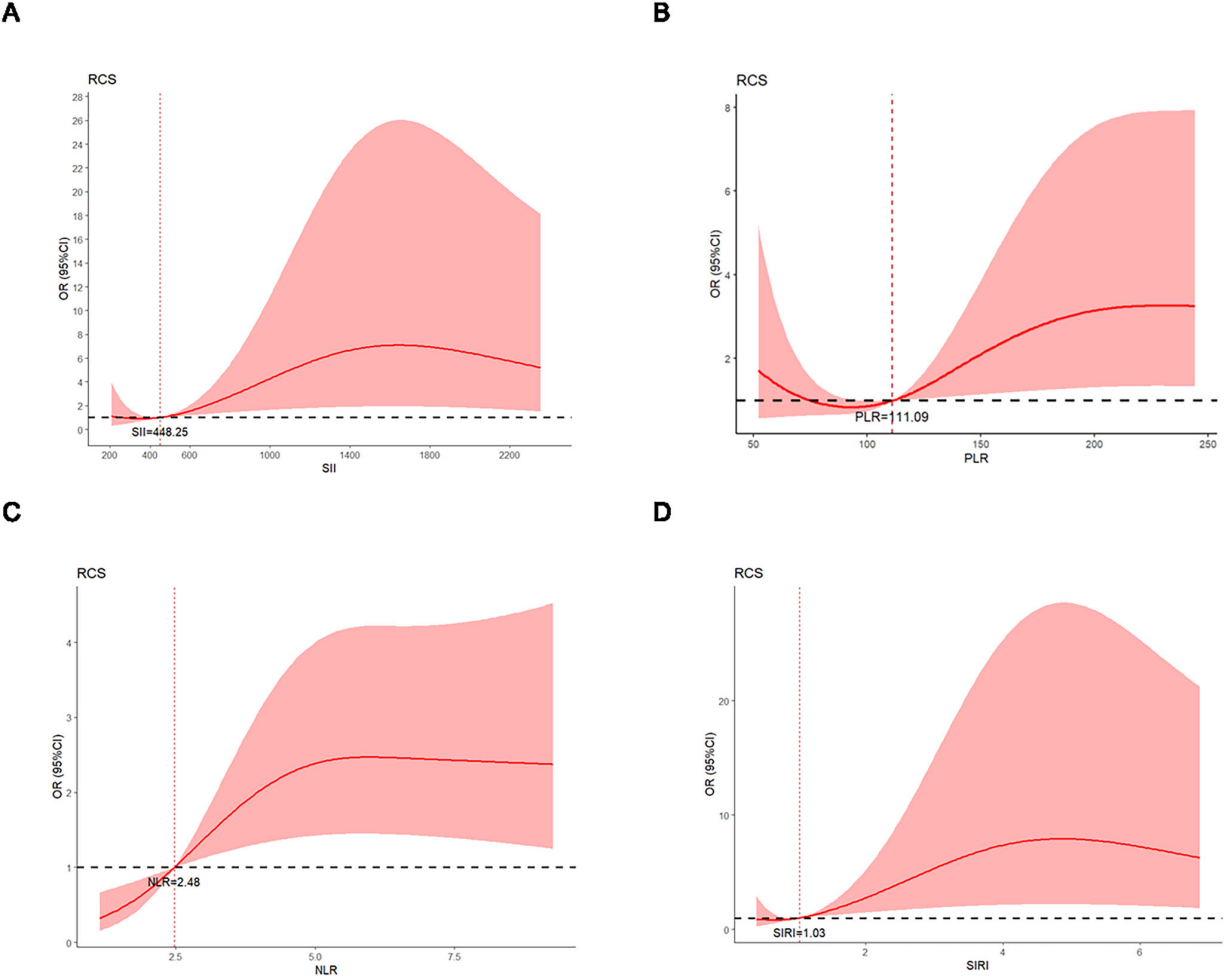
The restricted cubic spline plot of the association between the unfavorable outcomes and SII (A), PLR (B), NLR (C), and SIRI (D). NLR, neutrophil‐to‐lymphocyte ratio; PLR, platelet‐to‐lymphocyte ratio; SII, systemic immune‐inflammation index; SIRI, systemic inflammation response index.

The RCS curve illustrates the nonlinear relationship between SII, PLR, NLR, SIRI, and the 90‐day unfavorable Outcomes of AIS patients with intravenous thrombolysis (P for nonlinearity < 0.05, Figure 4). Of note, a threshold effect emerged, with a turning point observed at an SII value of 448.25, PLR value of 111.09, NLR value of 2.48, and SIRI value of 1.03.

### 3.6 Univariate and Multivariate Logistic Analysis of SII, PLR, NLR, and SIRI With 90‐Day Outcomes in AIS Patients With Intravenous Thrombolysis

During univariate regression analysis, the univariate logistic regression analysis indicated that gender, age, Atrial fibrillation, admission NIHSS‐scores, uric acid, neutrophil counts, albumin, SII, PLR, NLR, SIRI, and stroke etiology were associated with unfavorable outcomes at 90 day. In an exploratory multivariable logistic regression model adjusting for factors significant in univariate analysis, admission NIHSS score (OR = 1.156, 95% CI: 1.049–1.274), albumin (OR = 0.875, 95% CI: 0.774–0.989), and SII (OR = 1.004, 95% CI: 1.001–1.006) were retained as statistically independent association with 90‐day unfavorable outcomes (*p* < 0.05; refer to Table 4).

**TABLE 4:** Logistic regression models for unfavorable 90‐day outcomes.

Covariates	Univariate analysis		Multivariate analysis	
	OR (95% CI)	*p* value	OR (95% CI)	*p* value
Gender	0.471 (0.228–0.973)	0.04	0.677 (0.238–1.926)	0.46
Age	1.034 (1.005–1.065)	0.02	0.987 (0.946–1.028)	0.52
Atrial fibrillation	3.120 (1.121–8.682)	0.03	0.756 (0.104–5.517)	0.78
Admission NIHSS score, median (IQR)	1.215 (1.119–1.319)	0.00	1.156 (1.049–1.274)	0.02
Uric acid (µmol/L)	0.996 (0.991–1.000)	0.08	0.998 (0.992–1.003)	0.44
Neutrophil × 109/L, median (IQR)	1.160 (1.038–1.295)	0.01	1.330 (0.920–1.925)	0.13
Albumin	0.869 (0.789–0.957)	0.00	0.875 (0.774–0.989)	0.03
SII	1.005 (1.002–1.008)	0.01	1.004 (1.001–1.006)	0.02
PLR	1.002 (1.000–1.011)	0.07	0.999 (0.988–1.009)	0.79
NLR	1.090 (1.004–1.183)	0.04	0.810 (0.563–1.164)	0.25
SIRI	1.970 (1.278–2.229)	0.11	0.968 (0.698–1.342)	0.84
Stroke subtype (TOAST)				
LAA	1			0.20
SAA	0.254 (0.112–0.577)	0.00	0.32 (0.12–0.853)	0.02
CE	4.221 (1.092–16.319)	0.04	1.729 (0.16–18.674)	0.65
SAO	2.412 (0.142–40.823)	0.54	0.756 (0.006–101.924)	0.91
SUE	0.804 (0.078–8.285)	0.85	1.587 (0.119–21.175)	0.73

## 4 Discussion

In this retrospective cohort of AIS patients treated with intravenous thrombolysis, we found that elevated pre‐thrombolysis SII was independently associated with unfavorable 90‐day outcomes. SII demonstrated the highest specificity (92.0%) among the four inflammatory indices, and its discriminative ability was comparable to NLR (AUC 0.764 vs. 0.717, *p* = 0.392) but superior to PLR. RCS analysis further revealed nonlinear relationships between all four markers and outcome risk, with threshold effects observed at relatively low values. These findings underscore the potential role of baseline systemic inflammation in post‐thrombolysis prognosis.

Inflammatory biomarkers, including SII, PLR, NLR, and SIRI, may indicate systemic inflammation and immune response (R. H. Wang et al. 2023). In recent years, there has been an increasing focus on neuroinflammation, and various studies have confirmed the pivotal role of inflammatory mechanisms in the development and progression of ischemic stroke (Chamorro et al. 2016). The inflammatory response caused by neurological injuries and the cytokine release from immune cells leads to the production of anti‐inflammatory signals that inhibit cytokine production, ultimately preventing disease progression and infection (Johnston and Webster 2009).

Sex differences may also influence stroke outcomes, as females often present with different risk factor profiles and stroke subtypes (Torres‐Riera et al. 2025). In our cohort, although univariate analysis showed a higher proportion of females in the unfavorable outcome group, this association was attenuated after multivariable adjustment, possibly due to confounding by age and other comorbidities. Dedicated studies are needed to clarify sex‐specific prognostic effects.

Previous studies have demonstrated that higher SII, PLR, NLR, and SIRI are independently linked to the incidence, severity, and poor outcomes in stroke patients. Numerous studies have investigated the inflammatory response during the acute stage of cerebral infarction. These studies have consistently shown a close relationship between changes in inflammation biomarkers and ischemic stroke (Low et al. 2019). Neutrophils, platelets, and lymphocytes are essential components of the immune system and play a crucial role in responding to injuries, which are involved in the pathophysiology of vascular diseases (Ali et al. 2015; Adams et al. 2020). Previous research has indicated that higher levels of SII, PLR, NLR, and SIRI are independently associated with stroke incidence, severity, and poor outcomes (Yang et al. 2023; B. Zhu et al. 2018). We extended previous studies focusing on the relationship between these four indicators and intravenous thrombolysis in cerebral infarction. The RCS analysis revealed nonlinear relationships between all four inflammatory markers and the risk of unfavorable outcomes. Notably, the risk of an unfavorable outcome began to increase significantly once the SII exceeded 448.25, indicating this point as a potential threshold for heightened risk. However, these are exploratory, data‐driven findings and must not be misinterpreted as validated clinical thresholds. The disparity between this inflection point and the higher ROC cutoff further illustrates the difference between a level associated with initial risk elevation and one that robustly identifies high‐risk individuals. This value is substantially lower than the optimal ROC cutoff value of 1268.00, which was determined to best discriminate between outcome groups with high specificity. The disparity suggests that while the risk starts to rise at a lower SII level, a higher value is required to confidently predict a poor outcome in a clinical setting. Experimental studies have demonstrated that tPA administration can mobilize peripheral immune cells in animal models (Shi et al. 2021). Consistent with this, some clinical studies (Gong et al. 2021) have reported dynamic changes in neutrophil and lymphocyte counts following thrombolysis. Studies (Ören et al. 2022) also found that blocking the transport of lymphocytes and neutrophils in patients with intravenous thrombolytic therapy alleviated tPA‐induced cerebral hemorrhage. Interactions between lymphocytes and endothelial cells can lead to microvascular dysfunction and the release of inflammatory factors. This, in turn, can disrupt the blood‐brain barrier and cause neuronal cell death. Our findings add clinical observational evidence to this body of literature by demonstrating that pre‐existing systemic inflammation, as reflected by elevated SII, is independently associated with poorer recovery. However, whether this reflects a direct synergistic effect between thrombolysis and inflammation, or simply the prognostic importance of baseline inflammatory burden, cannot be determined from our data and requires dedicated mechanistic studies.

A recent study by Cao et al. (2024) in endovascular thrombectomy patients found that inflammatory indices measured on day 1 post‐procedure, but not admission values, were independently associated with outcomes, and that no admission variables were identified as independent risk factors. This contrasts with our findings in thrombolysis‐treated patients and highlights an important nuance: the prognostic value of admission inflammatory markers may differ by revascularization strategy. It is plausible that in thrombolysis—a pharmacological intervention that itself modulates systemic inflammation—baseline inflammatory status carries greater prognostic weight than in mechanical revascularization. This hypothesis, while speculative, warrants direct comparative studies.

This study provides a comparative and associative analysis of four inflammatory indices in a homogeneous cohort of AIS patients treated with intravenous thrombolysis. SII, NLR, PLR, and SIRI are four composite ratios in the combination of different inflammatory parameters, and they may be able to provide valuable insights into the immunological activities of ischemic stroke. Besides, these ratios can be calculated from blood cell counts, making them relatively accessible. Our results showed that these four indexes performed well in predicting AIS patients’ 90‐day poor neurological outcomes. A key finding of our comparative analysis is that only SII remained independently associated with the outcome after multivariable adjustment, while the associations for NLR, PLR, and SIRI were attenuated. This may be attributed to the fact that SII simultaneously incorporates platelet, neutrophil, and lymphocyte counts, potentially providing a more comprehensive reflection of the interplay between thrombosis and inflammation—a pathophysiological axis highly relevant to post‐thrombolysis outcomes.

While our study and several others (Li et al. 2025, 2024) have reported significant associations between inflammatory indices and post‐stroke outcomes, it is important to acknowledge that the literature is not entirely consistent. A recent meta‐analysis by X. Ma et al. (2026). Comparing SII and NLR found that NLR demonstrated a statistically significant association with poor outcome (OR = 1.26), whereas SII did not (OR = 1.00, *p* = 0.384). The pooled AUCs were also marginally higher for NLR (0.71 vs. 0.68), although the difference was not statistically significant. These findings stand in partial contrast to our results. Several factors may explain these discrepancies. First, the meta‐analysis by Ma et al. included heterogeneous stroke populations (both thrombolysed and non‐thrombolysed, AIS and overall ischemic stroke). Second, differences in the timing of blood sampling, outcome definitions (mRS > 2 vs. ≥ 3), and adjustment for confounders may contribute to variability in effect estimates. Third, the predictive performance of these indices may vary substantially by TOAST etiological subtypes, with NLR showing superior performance in large‐artery atherosclerosis and cardioembolic stroke, while SII performed more consistently across subgroups.

The purpose of this survey was to study the correlation between four indicators and the 90‐day outcomes. We analyzed the correlation coefficients and found that the SII had the strongest correlation. ROC analysis revealed that the SII exhibited the best specificity prediction of unfavorable outcomes, and multivariable logistic regression showed that the SII was an independent risk factor. A key objective of this study was to directly compare the associations of four inflammatory indices with 90‐day outcomes within a single, homogenous thrombolysis‐treated cohort. Our findings reveal a nuanced hierarchy of discriminative ability. SII and NLR demonstrated comparable discriminative performance (DeLong test: *p* = 0.392), with AUCs of 0.764 and 0.717, respectively. Heightened systemic inflammation, as indicated by an increased SII, may contribute to exacerbated tissue damage and impaired neurological recovery post‐thrombolysis. Chu et al. (2020) demonstrated that in acute ischemic stroke patients who are eligible for thrombolytic therapy, SII increases dynamically from the beginning of symptoms. The mechanistic underpinnings of this relationship may involve the dysregulation of immune responses, promotion of thrombotic processes, and exacerbation of neuroinflammatory cascades, ultimately influencing the efficacy of thrombolytic intervention.

 NLR is a useful predictor for assessing subclinical inflammation. It can also predict HT after ischemic stroke (Massiot et al. 2019; Zhang et al. 2020). Additionally, a high NLR and PLR could be linked to symptomatic internal carotid artery stenosis. A study by Nam and colleagues revealed that an increased NLR might indicate the possibility of stroke‐associated pneumonia (Huang et al. 2019). In addition, studies by Huang and his team have shown that elevated PLR levels are associated with post‐stroke depression. For SIRI, we used NLR to study the role of monocytes in AIS. There was early migration and infiltration of monocytes in AIS. Activated monocytes secrete VEGF and break the blood‐brain barrier, increasing blood vessel permeability. We found that for AIS patients with SIRI > 1.03, the incidence of 90‐day unfavorable outcomes would occur. Previous studies have shown that PLR can be used as a powerful biomarker to predict the prognosis of acute ischemic stroke. Altintas et al. (2016) found that when patients with acute ischemic stroke received endovascular therapy, the lower the PLR value, the better the clinical prognosis (mRS ≤ 2). However, few studies have investigated the association between PLR and outcomes in patients with intravenous thrombolysis. Our study found that PLR at admission was a predictor of 90‐day prognosis in stroke thrombolytic patients, and RCS results were analyzed when PLR = 111.09 was associated with the lowest risk of an unfavorable outcome. Xu et al. (2019) discovered that patients with stroke who were treated with intravenous thrombolysis showed a higher PLR that was independently linked to poor outcomes and death at 3 months. Previous studies (Sun et al. 2022) have shown that PLR cannot predict the risks of poor outcome in patients with acute ischemic stroke on admission, but it can predict them 24 h after rt‐PA.

The study findings supplement the roles of NLR, PLR, and SIRI in cerebrovascular disease and offer new concepts for clinical practice. In addition, our results also showed that all these inflammatory indexes affect the outcomes and may provide additional information in the short‐term outcomes. It is important to note that this study has some limitations. First, the data analyzed in this study was obtained retrospectively from a single medical center, particularly the relatively small number of patients with unfavorable outcomes (*n* = 96). which may introduce unmeasured confounding and restrict the external validity of the results. This also raises concerns regarding the events‐per‐variable ratio in our multivariable logistic regression analysis. Future prospective, multicenter studies are needed to confirm our findings. Second, in future research, it would be beneficial to monitor the indices throughout the entire treatment process rather than just relying on measurements taken before thrombolysis or at admission. Third, the exclusion of patients receiving bridging therapy restricts the generalizability of our findings to the broader population of AIS patients currently treated in comprehensive stroke centers. Future research should aim to validate our findings in larger, prospective multicenter cohorts and to explore whether serial measurements of inflammatory markers post‐thrombolysis provide additional prognostic value. Comparative studies across different stroke etiologies and revascularization modalities (e.g., bridging therapy) are needed to clarify the context‐dependent role of these indices. Moreover, sex‐specific analyses and mechanistic investigations into how systemic inflammation modulates the response to thrombolysis could inform personalized treatment strategies.

## 5 Conclusion

In this retrospective, exploratory analysis of AIS patients treated with intravenous thrombolysis, elevated pre‐thrombolysis SII was independently associated with unfavorable 90‐day outcomes. DeLong testing demonstrated that SII and NLR exhibited comparable discriminative ability, with no statistically significant difference; both significantly outperformed PLR. However, these findings are hypothesis‐generating only. Due to the modest discriminative ability, absence of validation, unmeasured confounding (including LVO status), and single‐center design, this study does not support any clinical application, risk stratification, or trial use of these indices.

## Author Contributions

**Xinping Bai**: conceptualization, writing – original draft, writing – review and editing. **Zhuo Cai**: software, investigation. **Juluo Chen**: conceptualization, writing – review and editing, methodology. **Xiangyu Wu**: software, investigation. Hao Cheng: investigation, software.

## Ethics Statement

All participants signed and provided written informed consent. This study was approved by the Research Ethics Committee at Fuyang People's Hospital ([2022]159).

## Funding

The authors have nothing to report.

## Conflicts of Interest

The authors declare no conflicts of interest.

## Data Availability

The datasets generated and analyzed during the current study are not publicly available due to data regulations but are available from the corresponding author on reasonable request.
